# Characterization of morphology and function of the ‘neo-atria’ after a modified Mustard operation

**DOI:** 10.21542/gcsp.2021.21

**Published:** 2021-10-30

**Authors:** Mohamed Nagy, Hatem Hosny, Amr El Sawy, Ahmed Mahgoub, Magdi H. Yacoub

**Affiliations:** 1 Biomedical Engineering and Innovation Laboratory, Aswan Heart Centre, Aswan, Egypt; 2 Cardiac Surgery Department, Aswan Heart Centre, Aswan, Egypt; 3 National Heart and Lung Institute, Imperial College, London, United Kingdom

## Abstract

**Background:** There is a pressing need to improve early and long-term results of the Mustard operation. A modification of the operation was introduced at the Aswan Heart Centre for this purpose which relies on creating new functional atria rather than the two rigid channels in the classical Mustard operation.

**Objectives:** To evaluate the morphology and function of the neo-atria, shortly after modified mustard operation for a ‘neglected’ patient with TGA, VSD and severe pulmonary hypertension.

**Methods:** A 6-year-old with neglected TGA, VSD and pulmonary hypertension presented with severe cyanosis, clubbing and haemoconcentration (Hb 22 g/dL), underwent the modified Aswan-Mustard operation (MAM) with rapid smooth postoperative recovery. Repeated 2D echograms and multi-slice CT scans, followed by 3D segmentation, were performed after the operation. The size, shape, and morphology of the neo-atria were measured and measurements of the patterns of instantaneous filling and emptying of the right and left ventricles were quantified.

**Results:** The neo-systemic venous atrium consisted of three components with a combined volume of 78 mL/m^2^, all of which contributed to the reservoir, conduit, and importantly contractile function of the neo-atrium. The pulmonary venous atrium consisted of two components with a combined volume of 66 mL/m^2^. These measurements were made at atrial end diastole. The volumes of the systemic venous and the pulmonary venous diminished to 51 and 54 mL/m^2^, respectively, at the end atrial systole - indicating relatively preserved contractile functions.

**Conclusion:** Following the modified Aswan-Mustard operation, neo-atrial function was relatively well preserved compared to the classical operation. The long-term results of these findings and their effects on quality of life need to be studied further.

## Introduction

The Mustard operation was modified by our group^1,2^ to improve the overall results of the operation by preserving the conduit, reservoir, and contractile functions^3^ of the neo-atria. These functions depend on the exact size, shape (morphology) and function of the neo-atria. Multislice computed tomography, combined with 3D image analysis, is a powerful tool for measuring these parameters. Here we describe the morphology and function of the neo-atria in a patient shortly after the modified Mustard operation for complex transposition of the great arteries (TGA).

## Patient and methods

A 6-year-old male child from Ethiopia was referred to the Aswan Heart Centre with a diagnosis of dextro-transposition of the great arteries (d-TGA), atrial septal defect (ASD), and ventricular septal defect (VSD). Although he was cyanotic since birth, his cardiac condition was diagnosed at the age of 4 years. The patient complained of shortness of breath and excessive sweating on mild exercise. Clinical assessment showed average build for age, cyanosis (SpO_2_ 72%), and blood haemoglobin level of 22 mg/dl, with normal motor and mental development. ECG showed normal sinus rhythm and right axis deviation. Echocardiography showed d-TGA (side-by-side great arteries), large ASD with bidirectional flow and a large inlet VSD with outlet extension.

Cardiac catheterization was performed on 100% oxygen only because he was severely cyanosed during anaesthesia (Table 1).

**Table 1:** Results from the hemodynamic study during cardiac catheterization.

Chamber	Pressure (mmHg)	Saturation %	pO_2_ (mmHg)
MPA	97/52/72	96	75
AO	108/85/95	90.9	73.6

The patient underwent the modified Aswan-Mustard (MAM) procedure and VSD closure with flap fenestrated patch. Intraoperative direct pressure measurements showed systolic aortic pressure of 75 mmHg and systolic pulmonary artery pressure of 47 mmHg.

The patient had a smooth and rapid postoperative ICU course, was extubated within 12 h, and discharged from ICU after 48 h - with normal sinus rhythm. Early post-operative echocardiography showed good biventricular function, laminar flow across both pulmonary and systemic venous baffles and bidirectional flow across the fenestrated VSD patch, mainly left-to-right. The total length of hospital stay was 5 days.

## CT segmentation

3D segmentation of both atria and ventricles was performed on CT scans using Mimics Innovation Suite (Materialise NV, Leuven, Belgium). This was followed by measurement of instantaneous volume changes of the atria during the cardiac cycle.

## Results

### Morphology of the neo-atria

**Figure 1.**
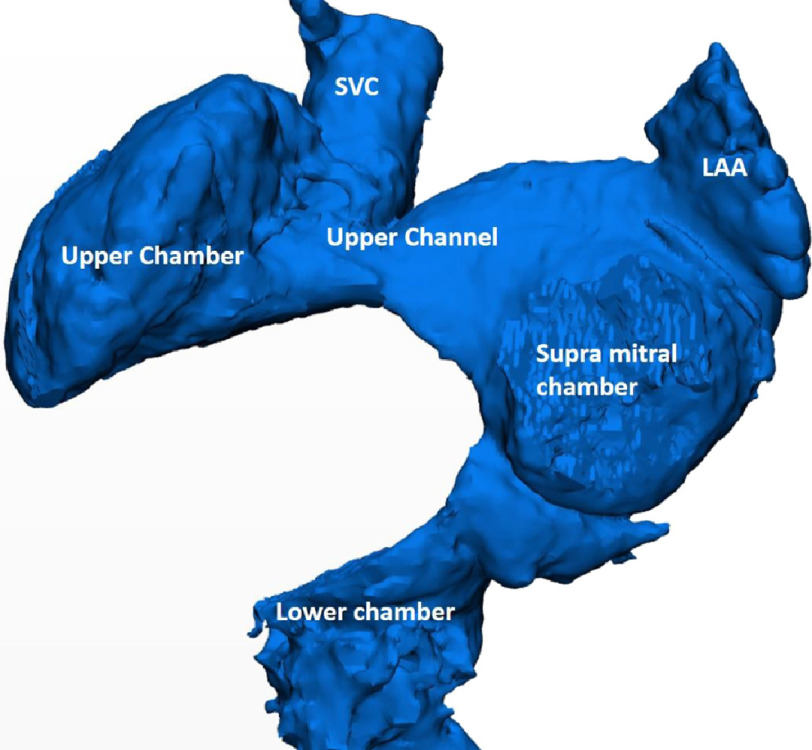
CT derived 3D reconstruction of the neo-systemic venous atrium showing the three components.

The systemic venous neo-atrium consisted of three compartments:

1. An upper chamber, which received the superior vena cava and included the sinoatrial node and the intact right atrial appendage (Figure 1).
2. A supra-mitral chamber, which received the left atrial appendage (LAA) and was connected to the upper chamber with a wide upper channel.
3. The lower chamber, which received the inferior vena cava and was connected to the supra mitral chamber with a wide, short channel.

**Figure 2.**
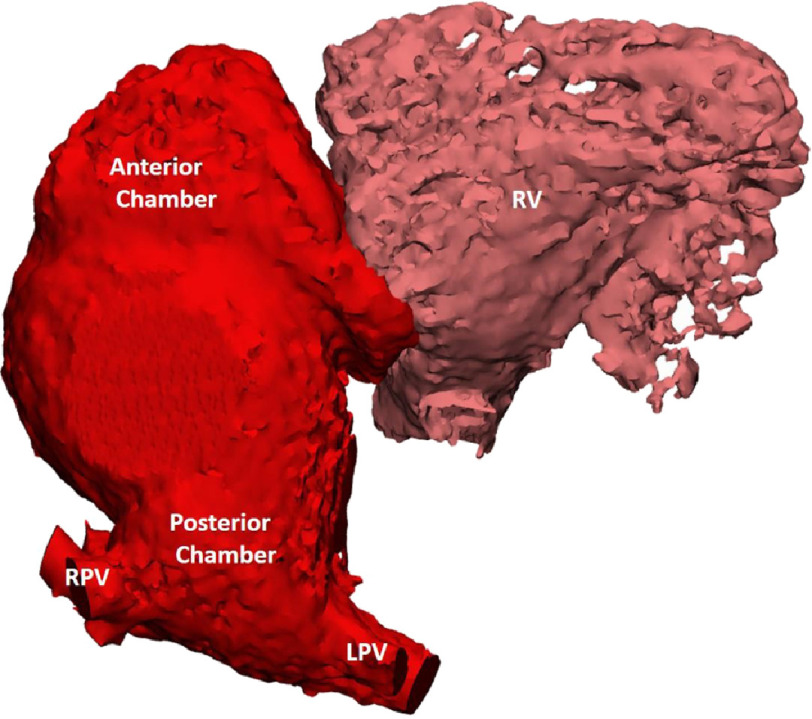
CT derived 3D reconstruction of the neo-pulmonary venous atrium showing the two components.

The pulmonary venous atrium consisted of two compartments—namely a posterior chamber receiving the four pulmonary veins, connected by a ‘wide waist’ to a much bigger anterior chamber. The latter is connected to the right ventricle through the tricuspid valve (Figure 2).

**Figure 3.**
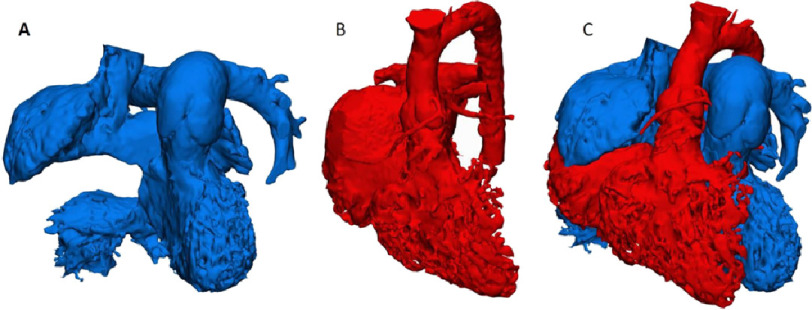
CT derived 3D reconstruction of systemic venous connections (A), pulmonary venous connections (B), combined systemic and pulmonary venous connections (C).

The overall appearance of the cardiac chambers and great vessels, following repair, is shown in Figure 3.

## Contractility of the neo-atria

### Systemic venous neo-atrium

At end atrial diastole, the volume of the upper chamber and channel measured 30 mL, the volume of the supra mitral combined with the left atrial appendage measured 17 mL, and the volume of the lower chamber and channel measured 8 mL. The combined volume (normalised over body surface area) was 78 mL/m^2^ and diminished to 51 mL/m^2^.

### Pulmonary venous neo-atrium

**Figure 4.**
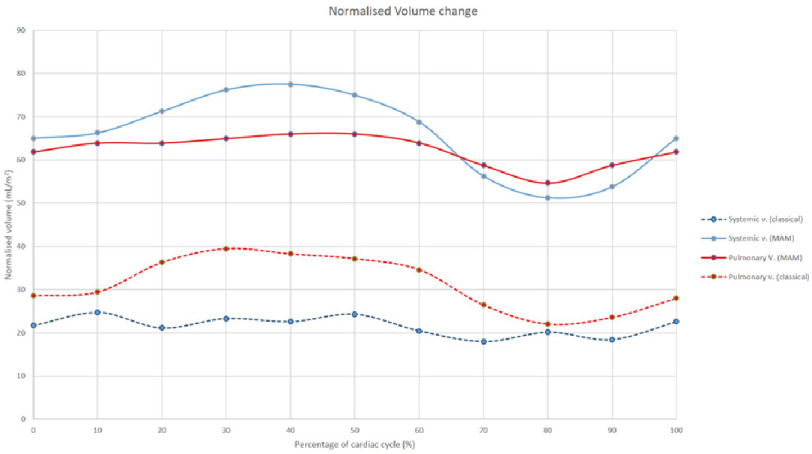
A curve showing instantaneous volume changes over cardiac cycle of the systemic venous and pulmonary venous neo-atria following MAM (solid curve) compared to the classical operation (dotted curve).

At end atrial diastole, the posterior chamber measured 13 mL and the anterior chamber measured 50 mL. The combined volume (normalised over body surface area) was 66 mL/m^2^ and diminished to 54 mL/m^2^. Both results are summarised in Figure 4 and compared to the classical Mustard operation.

## Discussion

The Mustard operation produces immediate and dramatic improvements in arterial oxygenation and general clinical condition of patients with TGA. However, these patients continue to have several limitations when compared to normally healthy children^4^. These limitations include; lower exercise capacity, inability to increase stroke volume on exercise or in response to infusion of inotropic agents, and importantly, lower long-term survival rates.

Such limitations are thought to be due to the inability of the right ventricle to serve the systemic circulation, in the longer term due to inherent impaired “power production” or other factors, such as impaired filling due to absence of atrial function, which is replaced by two rigid tubes in the classical Mustard operation.

Recent investigations have shown a preserved inotropic state^5^. In addition, impaired ventricular filling following inflow correction has been documented^6,7^.

## Lessons learned

1. Patients with advanced (neglected) TGA derive immediate benefit after the modified Aswan-Mustard (MAM) operation.
2. Recovery is more rapid than with classical Mustard operation, due to the less traumatic nature of the procedure.
3. Severe pulmonary hypertension can regress after the MAM operation.
4. In patients with TGA, large VSD, and pulmonary hypertension, closure of the VSD using a ‘valvular’ fenestrated patch could be beneficial.
5. All three components of the atria are restored (to a large degree) after the MAM operation.
6. The MAM operation avoids injury to conducting tissue, or its blood supply. This can reduce the incidence of early - and possibly, late - arrhythmias.
7. The influence of restoration of neo-atrial functions on quality of life and long-term survival after the MAM operation needs to be studied.
